# Supplementary material for: Structural basis for C-degron selectivity across KLHDCX family E3 ubiquitin ligases
Source: Nat Commun. 2024 Nov 15;15:9899. doi: 10.1038/s41467-024-54126-z (PMC11568203; doi:10.1038/s41467-024-54126-z)
Supplement: Supplementary file 1 — Supplementary Information [file 41467_2024_54126_MOESM1_ESM.pdf]

Supplementary Information:

Structural basis for C-degron selectivity across KLHDCX-family E3 ubiquitin ligases

Scott, Chittori, et al.

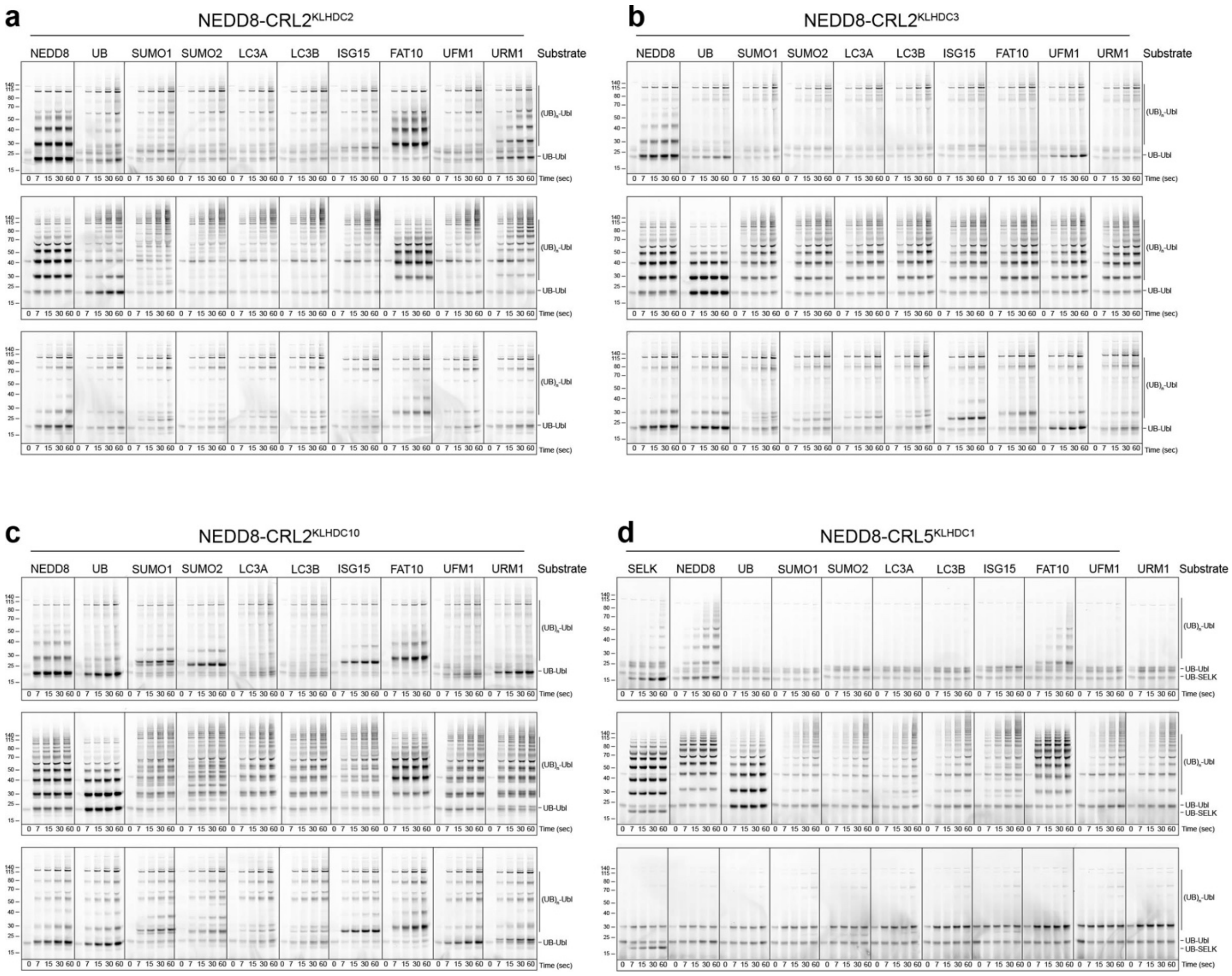

Supplemenetary Figure 1. Ubiquitylation of Gly terminating substrates by the KLH DCX family.

a. Fluorescent scan of gel from pulse-chase assay monitoring the ubiquitylation of UB or the indicated Gly terminating substrates with monomeric $CRL2^{KLHDC2}$ by UBE2D2 (top panel), UBE2R2 (middle panel), or UBE2L2/ARIH1 (bottom panel). All reactions were carried out with the respective UCE pulse-loaded with WT UB. Reactions with UBE2D2 and UBE2R2 were carried out at RT with pH =7.5. Reactions with UBE2L3/ARIH1 were performed at 0 °C with pH = 7.5. Samples were quenched at the indicated times with 2X SDS-PAGE sample buffer supplemented with 50 mM DTT to reduce the UCE~UB thiolester conjugate to aid in product quantifications. **b.** Same as **(a)**, but with monomeric $CRL2^{KLHDC3}$. **c.** Same as **(a)**, but with $CRL2^{KLHDC10}$. **d.** Same as **(a)**, but with $CRL5^{KLHDC1}$. All panels are representative images from n=2 independent experiments. Source data are provided as a Source Data file.

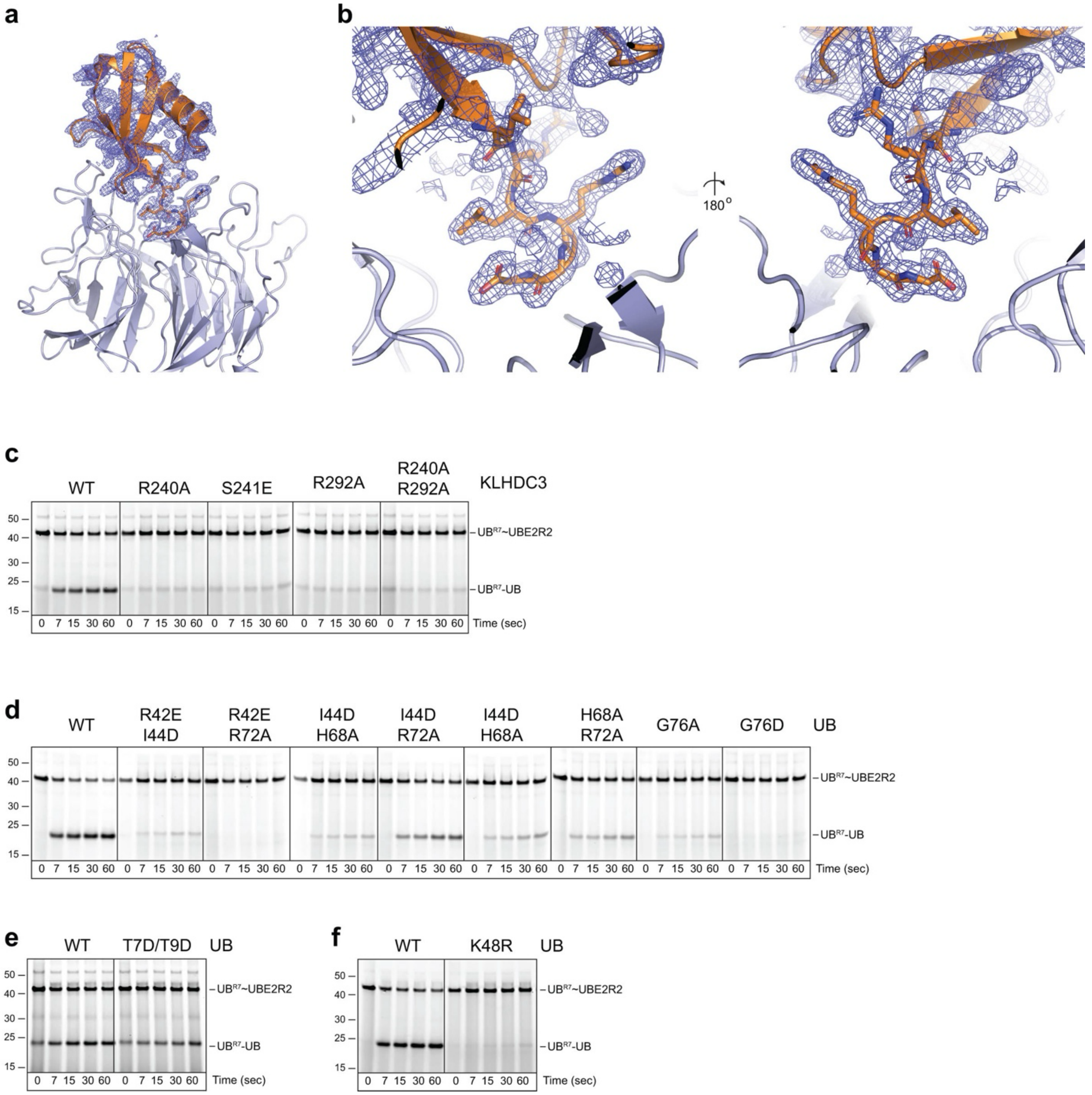

Supplementary Figure 2. Structure of UB bound to KLHDC3

a. 2Fo-Fc density at 1 σ surrounding UB (orange, cartoon, residues from UBs C-terminal degron are shown in sticks) bound to KLHDC3 (light blue, cartoon). **b.** Same as **(a)** but zoomed in to the KLHDC2 C-degron binding pocket. **c.** Fluorescent scan of gel from pulse-chase assay monitoring UBE2R2 mediated ubiquitylation of UB by WT or the indicated mutants of momeric KLHDC3. **d.** Same as **(c)** but monitoring ubiquitylation of WT UB or the indicated UB mutants by WT monomeric CRL2^{KLHDC3}. **e.** Same as **(d)**. **f.** Same as **(d)**. All panels are representative images from n=2 independent experiments. Source data are provided as a Source Data file.

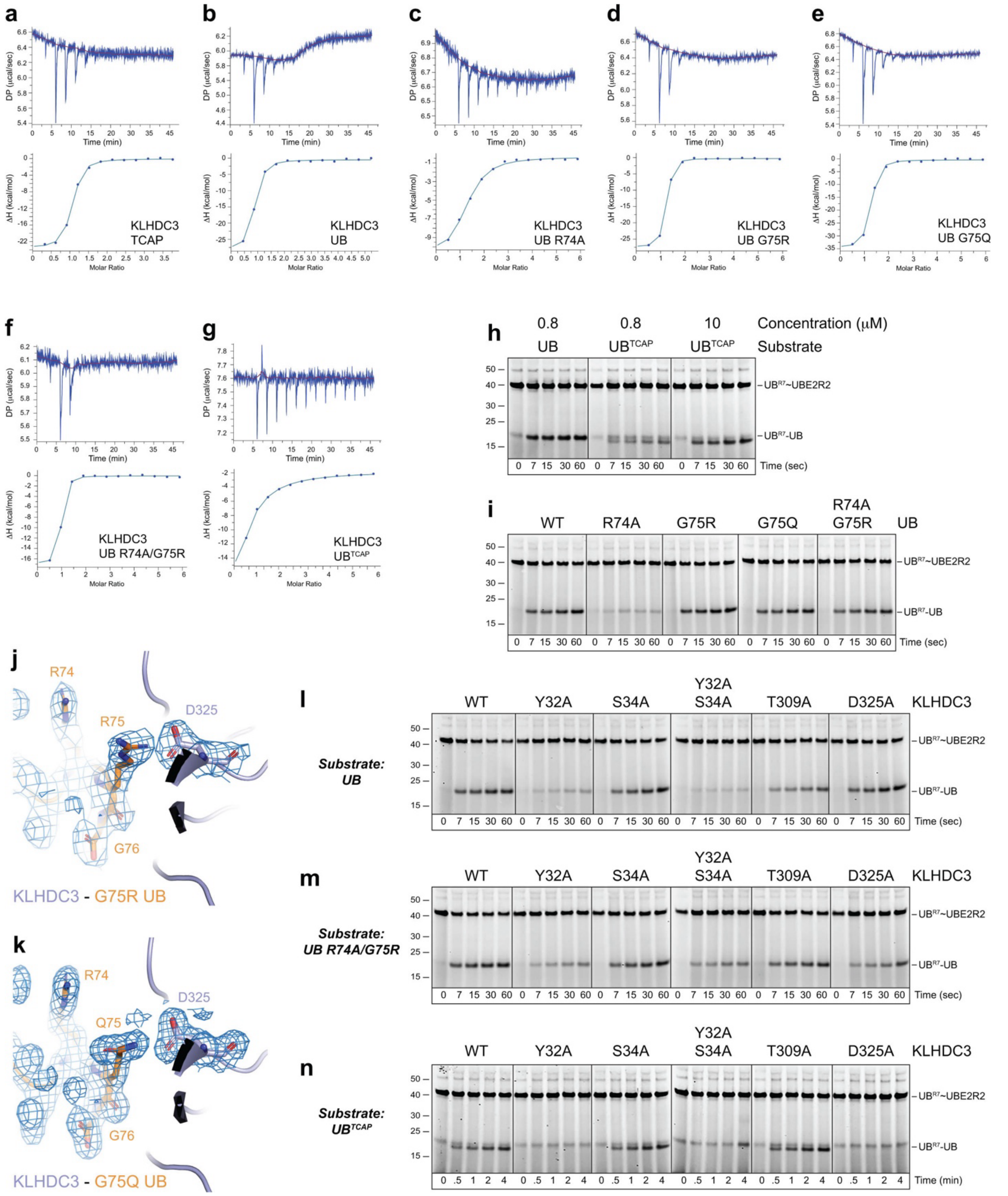

Supplementary Figure 3. Characterization of KLHDC3s penultimate binding pocket. a. Isothermal titration calorimetry (ITC) monitoring binding of TCAP peptide to monomeric KLHDC3 (top panel) and Isotherm fit of data (bottom panel) **b.** Same as **(a)**, but for UB binding to monomeric KLHDC3. **c.** Same as **(a)**, but for R74A UB binding to monomeric KLHDC3. **d.** Same as **(a)**, but for G75R UB binding to monomeric KLHDC3.

e. Same as **(a)**, but for G75Q UB binding to monomeric KLHDC3. **f.** Same as **(a)**, but for R74A/G75R UB binding to monomeric KLHDC3. **g.** Same as **(a)**, but for UB^{TCAP} binding to monomeric KLHDC3. **h.** Fluorescent scan of gel from pulse-chase assay monitoring the UBE2R2 mediated ubiquitylation of UB or UB^{TCAP} substrates at the indicated concentrations by monomeric $CRL2^{KLHDC3}$. **i.** Same as **(g)** but monitoring ubiquitylation of WT UB or the indicated UB mutants by monomeric $CRL2^{KLHDC3}$. **j.** 2Fo-Fc density at 1 σ surrounding G75R UB (orange, cartoon, residues from UBs C-terminal degron are shown in sticks) bound to monomeric KLHDC3 (light blue, cartoon, D325 is shown in sticks). **k.** same as (**j**) but for G75Q UB. **l.** Same as **(g)** but monitoring ubiquitylation of WT UB by WT or the indicated monomeric mutants of KLHDC3**. m.** Same as **(g)**, but with R74A/G75R mutant of UB. **n.** Same as **(g)**, but with UB^{TCAP}. All panels are representative images from n=2 independent experiments. Source data are provided as a Source Data file.

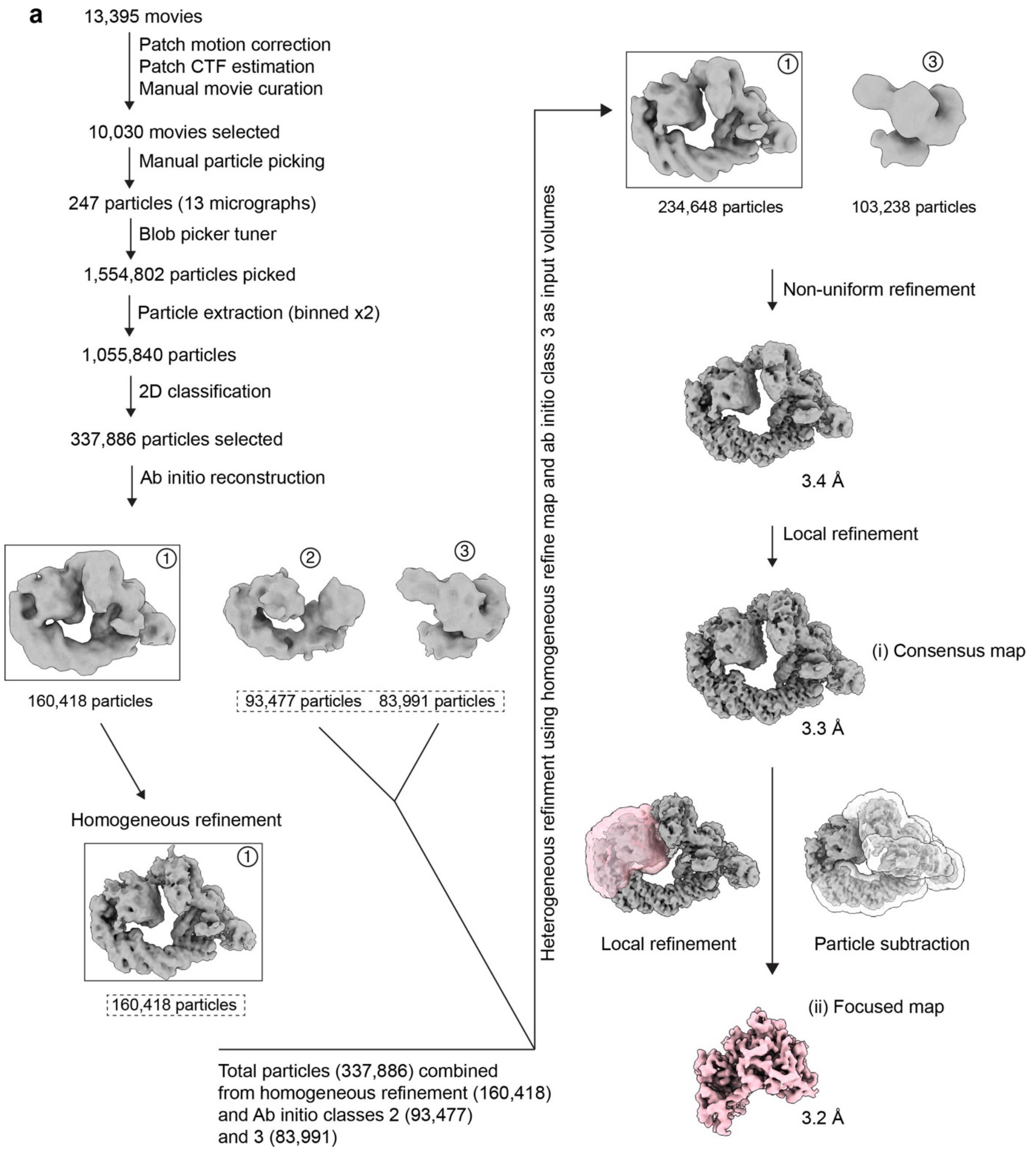

Supplementary Figure 4. Cryo-EM image processing workflow of ARIH1-UB-UB-neddylated-CRL2^{KLHDC10} complex.

a. Overview of the data processing steps used to generate the ARIH1-UB-UB-neddylated-CRL2^{KLHDC10} consensus map and KLHDC10-EloB/C focused map presented in this study. The ab-initio model corresponding to the ARIH1-UB-UB-neddylated-CRL2^{KLHDC10} complex **(class 1)** was homogenously refined and inverted to produce a right-handed structure. The final consensus map resolved at 3.3 Å **(ii)** was then utilized to resolve the KLHDC10-EloB/C component at 3.2 Å **(iii)** through particle subtraction and focused refinement. The masks applied to the consensus reconstruction for particle subtraction and focused refinement are shown in white and pink, respectively.

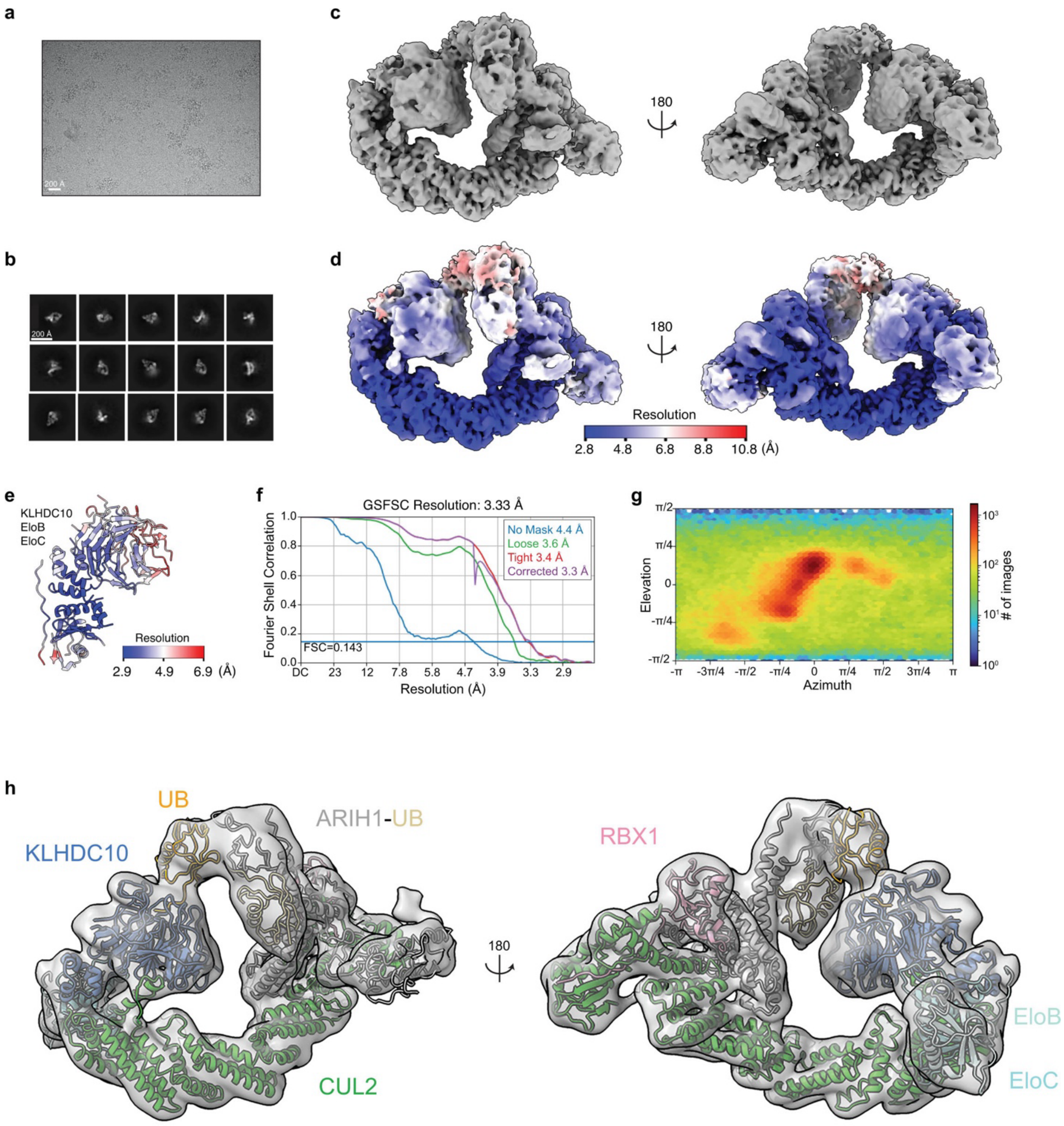

Supplementary Figure 5. Cryo-EM image processing analysis of ARIH1-UB-UB-neddylated-CRL2^{KLHDC10} consensus map.

a. Representative micrograph of ARIH1-UB-UB-CRL2^{KLHDC10} complex. **b.** Representative 2D classes showing multiple orientations. **c.** 180º rotated views of the final consensus 3D reconstruction. **d.** 180º rotated views of the final consensus map colored using local resolution values. **e.** KLHDC10-EloB/C model colored using the local resolution estimates of the final consensus map. **f.** Fourier shell correlation (FSC) curves of the final reconstruction. **g.** Angular

distribution of the particles used in the final reconstruction. **h.** Model of the full complex fitted into the low-pass filtered consensus map. Structural coordinates from EloB/C-CUL2-RBX1 (cyan,pale cyan, green, and light pink respectively;5N4W.pdb), AIRH1-UB, (gray and wheat respectively;7B5M.pdb and 7B5N.pdb), UB-KLHDC10 (orange and marine; 1UBQ.pdb and an AlphaFold model of KLHDC10) were bulk fit into the EM density in ChimeraX

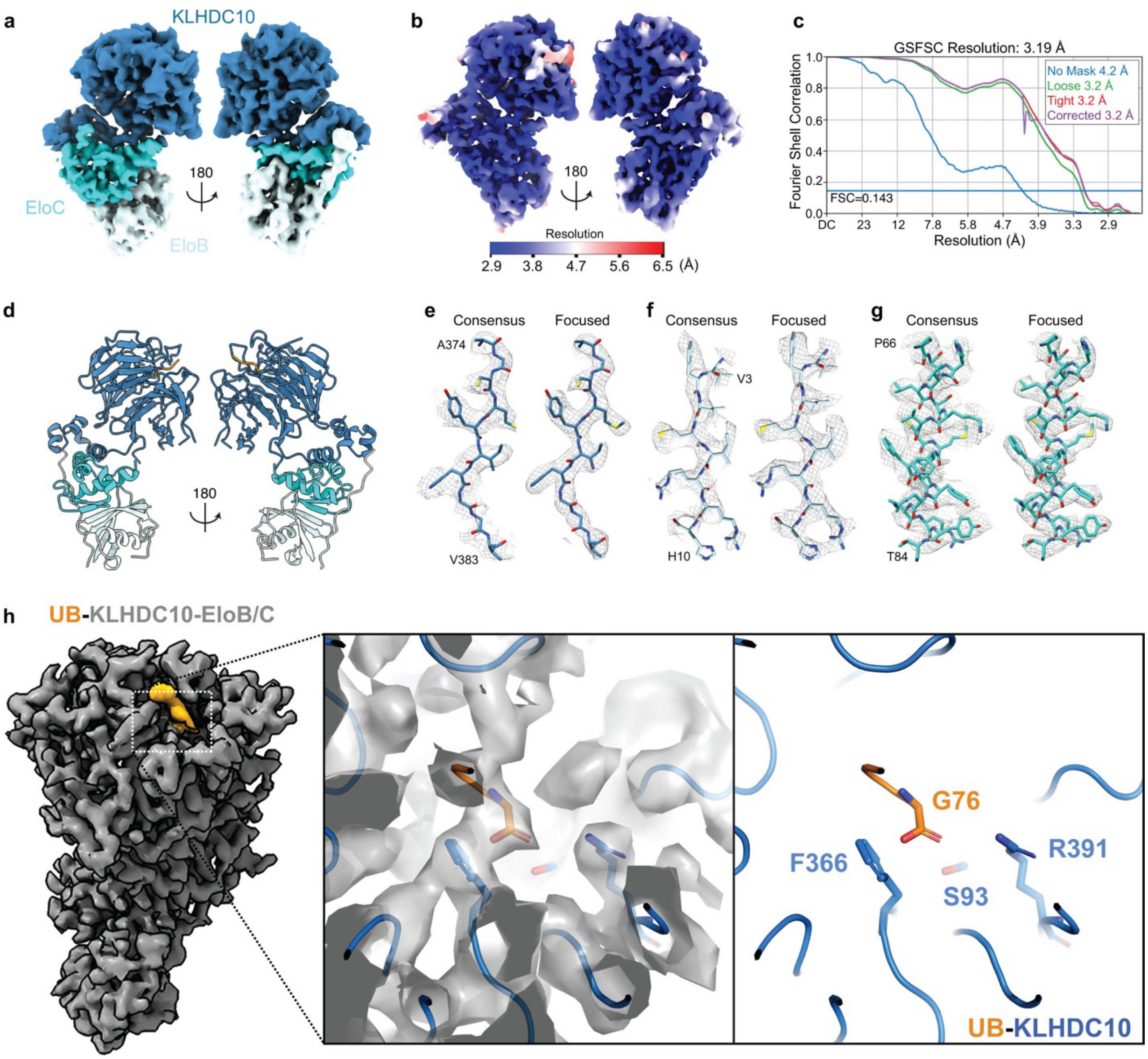

Supplementary Figure 6. Cryo-EM image processing and model building analysis of KLHDC10-EloB/C focused map.
a. 180º rotated views of the final focused map, colored by individual protein components (KLHDC10 in blue, EloB in light cyan, EloC in cyan). **b.** same as **(a)** but colored by local resolution values. **c.** Fourier shell correlation (FSC) curves for the final focused 3D reconstruction. **d.** Model of the KLHDC10-EloB/C complex with the ubiquitin c-degron bound to KLHDC10, using the same color scheme as in (**a**), with the ubiquitin c-degron highlighted in orange. **e-g.** Representative regions of the individual components—KLHDC10 **(e)**, EloB **(f)**, and EloC **(g)** are displayed to highlight the quality of their fitting within both the consensus map (right panels) and the focused map (left panels). **h.** Surface representation (left) of the focused map (gray) highlighting the density for ubiquitins c-degron (orange). Zoomed in view (middle) of the density surrounding Gly76 of UB (orange, sticks) and KLHDC10's FSR motif (sticks, marine).

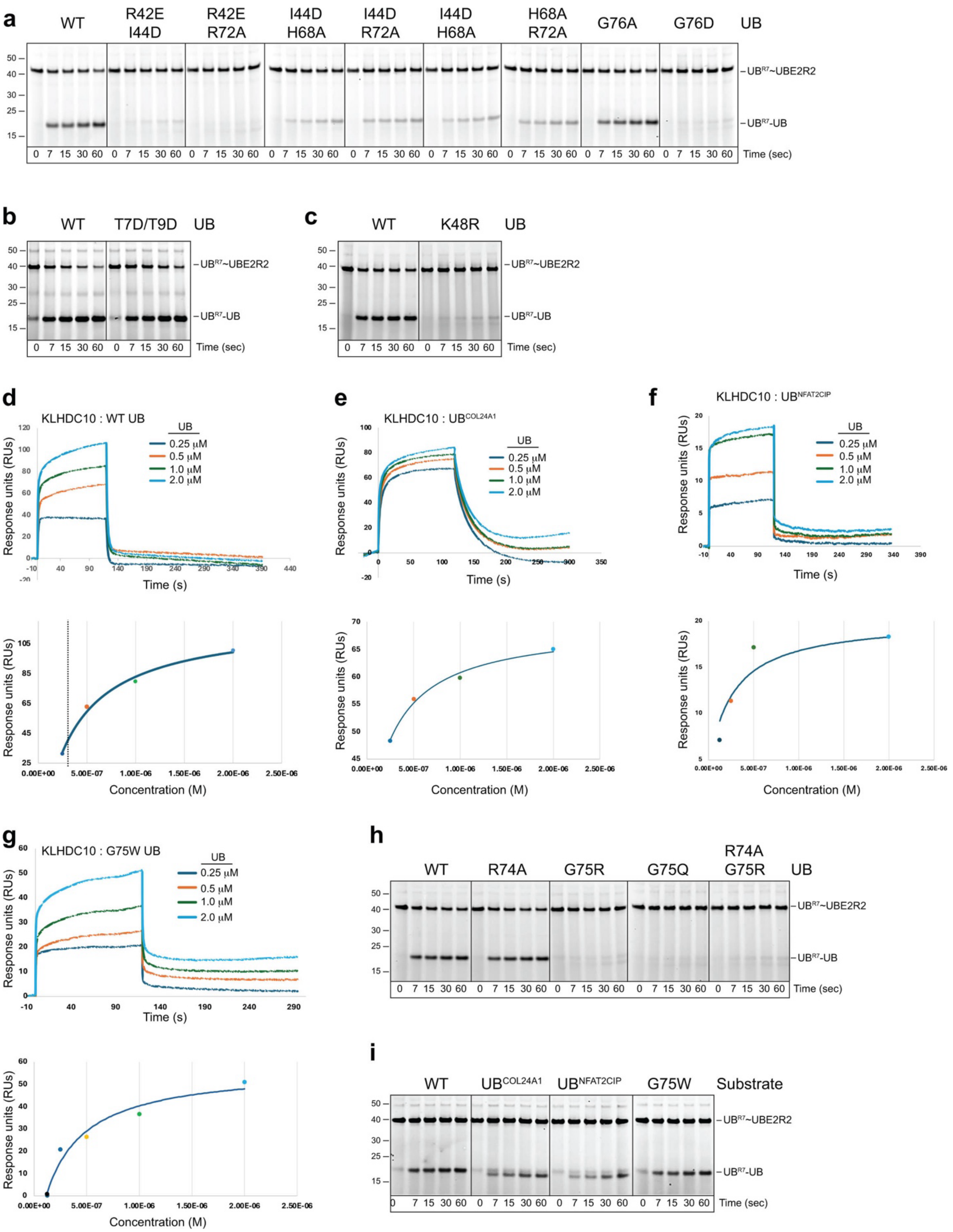

Supplementary Figure 7. Characterization of KLHDC10s penultimate binding pocket. **a.** Fluorescent scan of gel from pulse-chase assay monitoring the UBE2R2 mediated ubiquitylation of WT UB or the indicated UB mutants by CRL2^{KLHDC10}. **b.** Same as **(a)**. **c.**

Same as **(a)**. **d.** Surface plasmon resonance traces for binding of WT UB to KLHDC10 (top panel). Fit of SPR data from the top panel is shown (bottom panel). Shown is representative data from n=2 independent experiments. **e.** Same as **(d)**, but for $UB^{COL24A1}$ binding to KLHDC10. **f.** Same as **(d)**, but for $UB^{NFAT2CIP}$ binding to KLHDC10. **g.** Same as **(d)**, but for G75W UB binding to KLHDC10. **h.** Same as **(a)**. **i.** Same as **(a)**. All panels are representative images from n=2 independent experiments. Source data are provided as a Source Data file.

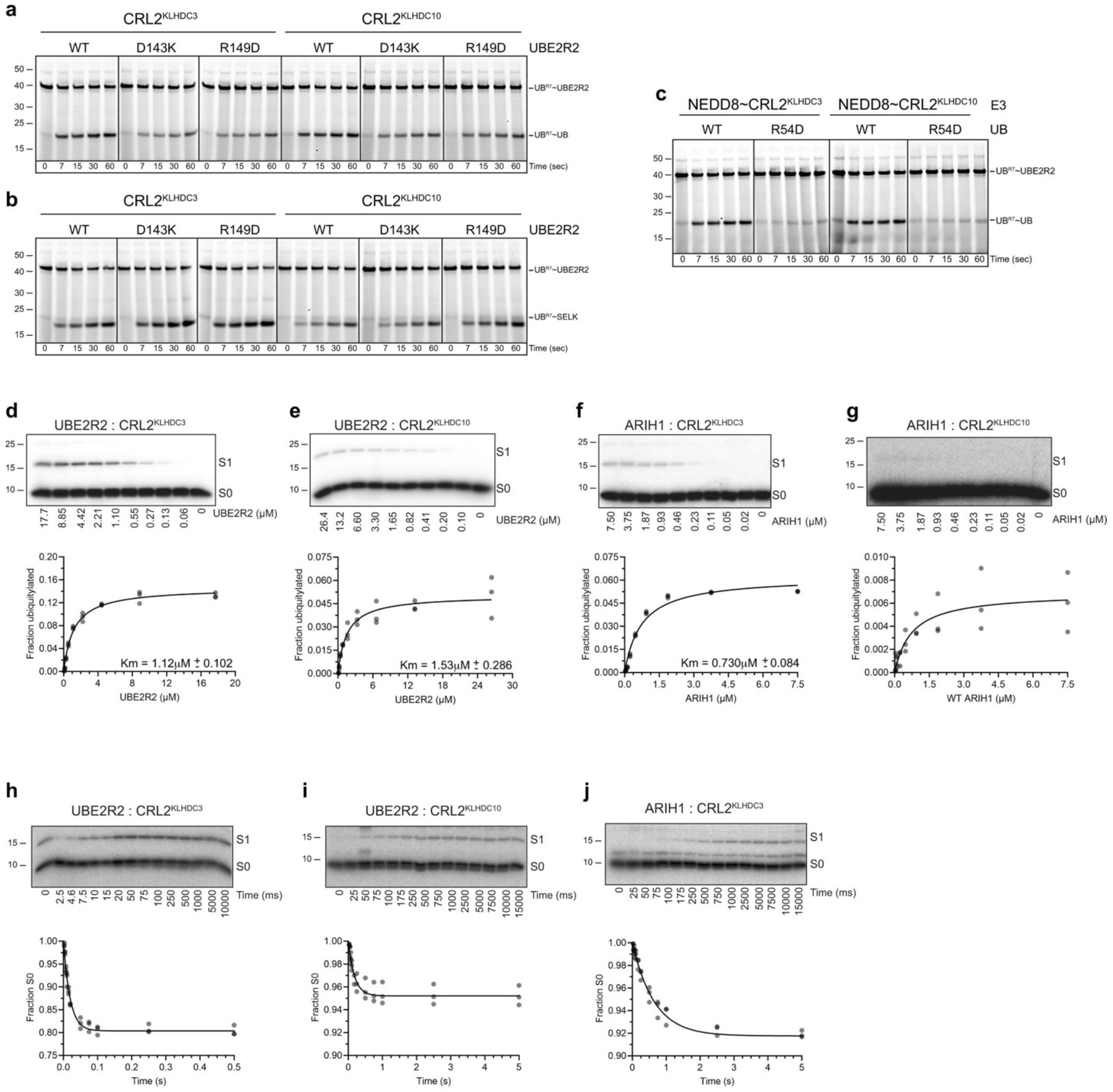

Supplementary Figure 8. CRL2^{KLHDC3} and CRL2^{KLHDC10} promote millisecond ubiquitylation of UB.

a. Fluorescent scan of gel from pulse-chase assay monitoring the UBE2R2 or indicated UBE2R2 mutants mediated ubiquitylation of UB or SELK by monomeric CRL2^{KLHDC3}. **b.** Same as **(a)**, but with CRL2^{KLHDC10}. **c.** Fluorescent scan of gel from pulse-chase assay monitoring the UBE2R2 mediated ubiquitylation of UB or the R54D UB acceptor mutant by monomeric CRL2^{KLHDC3} or CRL2^{KLHDC10}. **d.** Autoradiogram showing UB-UB formation for reactions containing UB, monomeric CRL2^{KLHDC3}, and increasing concentrations of UBE2R2. The gel migrations of radiolabeled UB (S0) and UB-UB product (S1) are shown (top). The graph shows the fraction of UB-UB product formed as a function of UBE2R2 concentration (bottom). The data were fit to the Michaelis-Menten model (GraphPad Prism software v9) to estimate the K_m of UBE2R2-mediated ubiquitylation complex. The autoradiogram is representative of triplicate technical replicates. **e.** Same

as **(d)**, but for CRL2^{KLHDC10}. **f.** Same as **(d)**, but for monomeric CRL2^{KLHDC3} and increasing concentrations of ARIH1. **g.** Same as **(d)**, but for CRL2^{KLHDC10} and increasing concentrations of ARIH1. **h.** Autoradiogram (top) showing UB-UB formation for pre-steady state reactions with UBE2R2 and monomeric CRL2^{KLHDC3}. Graphs (bottom) from pre-steady state kinetic studies showing the fraction of UB substrate remaining as a function of time with monomeric CRL2^{KLHDC3}. Data were fit to an analytical closed-form solutions model in Mathematica. Datapoints from triplicate technical replicates are shown. **i.** Same as **(h)** but for UBE2R2 and CRL2^{KLHDC10}. **j.** Same as **(h)** but for UBE2L2/ARIH1 and CRL2^{KLHDC3}. Source data are provided as a Source Data file.

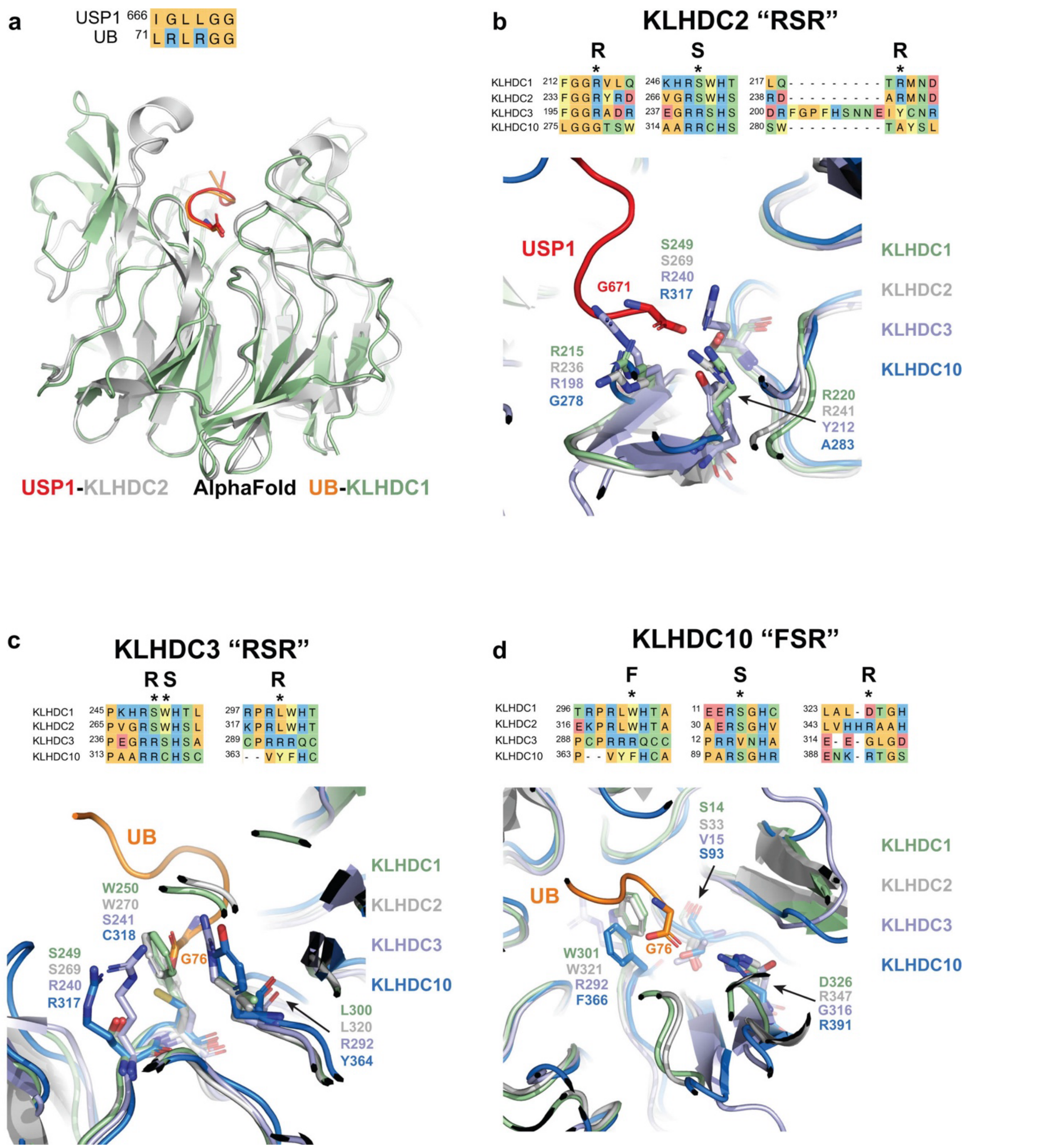

Supplementary Figure 9. Sequence divergence of R/F-S-R motifs across Kelch bla des 4-6 from the KLHDCX family.

a. Sequence alignment of the C-terminal 6 residues of the USP1 and UB C-degrons (top). Structural superposition (bottom) of KLHDC2 (gray, cartoon,) bound to USP1 (red, cartoon, G671 in sticks) and an AlphaFold 3 model of KLHDC1 (pale green, cartoon) bound to the UB C-terminus (orange, cartoon, G76 in sticks). **b.** Sequence alignment of KLHDC1, KLHDC2, KLHDC3, and KLHDC10 surrounding the RSR motif from KLHDC2s blade 4 (top). Structural superposition of an AlphaFold 3 model of KLHDC1 (pale green, cartoon, residues corresponding to KLHDC2s RSR motif

are shown in sticks), KLHDC2 (gray, cartoon, residues corresponding to KLHDC2s RSR motif are shown in sticks), KLHDC3 (light blue, cartoon, residues corresponding to KLHDC2s RSR motif are shown in sticks), and KLHDC10 (marine, cartoon, residues corresponding to KLHDC2s RSR motif are shown in sticks). **c.** same as (**b**) but for KLHDC3s RSR motif. **d.** same as (**b**) but for KLHDC10s FSR motif.

Supplementary Table 1. Data collection and refinement statistics for UB-KLHDC3-EloB/C crystal structures.

	UB-KLHDC3-EloB/C	G75R UB-KLHDC3-EloB/C	G75Q UB-KLHDC3-EloB/C
**PBD accession code**	9D1I	9D1Y	9D1Z
**Data Collection**			
Space group	$C222_1$	$C222_1$	$C222_1$
Cell dimensions			
a, b, c (Å)	114.71, 121.44, 153.142	114.56, 121.54, 152.95	114.95, 120.87, 152.95
α, β, γ (°)	90.0, 90.0, 90.0	90.0, 90.0, 90.0	90.0, 90.0, 90.0
Resolution range (Å)[a]	38.29-2.00	45.85-2.56	45.95-1.88
R-merge	0.107 (0.590)	0.156 (0.830)	0.099 (0.838)
r-pim	0.035 (0.202)	0.053 (0.276)	0.035 (0.310)
$CC_{1/2}$	0.996 (0.974)	1.006 (0.958)	0.997 (0.939)
$I / \sigma I$	18 (2.5)	10 (1.8)	23.5 (1.9)
Completeness (%)	99.8 (98.3)	100 (100)	100 (100)
Redundancy	4.6 (4.0)	8.4 (8.7)	8.1 (7.4)
**Refinement**			
Resolution (Å)	38.29-2.00	45.85-2.60	43.38-1.88
No. Reflections	70,046	32,925	84,717
R_{work}/R_{free}	0.1678/0.1894	0.1741/0.2121	0.1681/0.1896
No. atoms	5532	5343	5747
Protein	4912	4969	5031
Ligand	6	12	38
Water	613	362	678
**B-factors:**			
Protein	42.28	46.58	38.98
Ligand	35.57	47.79	43.27
Water	45.72	40.43	41.52
**R.m.s deviations:**			
Bond lengths (Å)	0.007	0.002	0.007
Bond angles (°)	0.89	0.21	0.99
**Ramachandran stats:**			
Ramachandran favored	98.06	97.74	97.78
Ramachandran allowed (%)	1.94	2.26	2.22
Ramachandran outliers (%)	0.0	0.0	0.0

Supplementary Table 2. Binding parameters for ITC studies with KLHDC3.

Sample cell	Syringe	K_d	ΔH	ΔG	-TΔS	N
KLHDC3	$TCAP^{Peptdie}$	224 +/- 20	-20.4 +/- 0.28	-8.9 +/- 0.3	11.5 +/- 0.6	0.95 +/- 0.08
KLHDC3	UB	321 +/- 55	-28.1 +/- 0.71	-8.8 +/- 0.7	19.3 +/- 0.8	0.71 +/- 0.02
KLHDC3	R74A UB	1460 +/- 202	-11.0 +/- 0.57	-7.9 +/- 0.4	3.10 +/- 0.6	1.1 +/- 0.02
KLHDC3	G75R UB	82 +/- 13	-27.1 +/- 0.34	-9.6 +/- 0.6	17.6 +/- 0.9	1.0 +/- 0.08
KLHDC3	G75Q UB	168 +/- 18	-34.3 +/- 0.49	-9.2 +/- 0.4	25.3 +/- 0.8	1.1 +/- 0.02
KLHDC3	R74A/G75R UB	94 +/- 16	-16.9 +/- 0.28	-9.5 +/- 0.5	7.42 +/- 0.2	0.79 +/- 0.07
KLHDC3	UB^{TCAP}	> 6000	ND	ND	ND	ND

Supplementary Table 3: Cryo-EM data collection and refinement statistics for ARIH1-UB-UB-CRL2^{KLHDC10}.

	**ARIH1-UB-UB-CRL2^{KLHDC10} (consensus)**	**KLHDC10-EloB/C (focused)**
EMDB ID	EMD-46644	EMD-46645
PDB ID		9D8P
**Data collection and image processing**		
Microscope	Titan Krios G3i	
Voltage (kV)	300	
Detector	K3	
Energy filter slit width (eV)	20	
Nominal magnification	130,000x	
Pixel size (Å)	0.6485	
Dose rate (e^-/px/s)	13.95	
Exposure time (s)	1.2	
Electron dose (e^-/Å^2)	~40	
Fractions	40	
Defocus range (μm)	-0.6 to -2.25	
Movies	13,395	
Initial particles	1,554,802	
Particles after 2D	337,886	
Particles in final 3D	234,648	
Symmetry	C1	
Overall map resolution (Å)	3.33	3.19
FSC threshold	0.143	0.143
**Model building and structure refinement**		
Chain/Residues/Atoms		4/537/4073
Model vs. Data CC_mask		0.83
d FSC model (0/0.143/0.5) (Å)		3.05/3.11/3.28
Rama-Z score		0.36
Ramachandran favored (%)		97.88
Ramachandran outliers (%)		0.00
Rotamers outlier (%)		0.00
RMSD Bond angles (°)		0.479
RMSD Bond lengths (Å)		0.003
Clashscore		9.78
MolProbity score		1.54
EMRinger score		3.38

FSC, Fourier shell correlation, CC, Correlation coefficient; RMSD, root mean square deviation

Supplementary Table 4. Binding parameters for SPR studies with KLHDC10

Analyte	K_d (nM)	Chi^2 (RU2)	Rmax
WT UB	356 +/- 37	12.7 +/- 11	93.6 +/- 63.9
$UB^{COL24A1}$	226 +/- 56	0.97 +/- 0.76	23.6 +/- 15.8
$UB^{NFAT2CIP}$	392 +/- 140	17.1 +/- 12.1	26.5 +/- 9.5
G75W UB	409 +/- 150	12.2 +/- 8.7	59.3 +/- 23.1
