# Supplementary material for: Structural basis for C-degron selectivity across KLHDCX family E3 ubiquitin ligases
Source: Nat Commun. 2024 Nov 15;15:9899. doi: 10.1038/s41467-024-54126-z (PMC11568203; doi:10.1038/s41467-024-54126-z)
Supplement: Supplementary file 2 — Reporting Summary [file 41467_2024_54126_MOESM2_ESM.pdf]

nature portfolio

Corresponding author(s): Brenda A. Schulman

Last updated by author(s): Oct 13, 2024

# Reporting Summary

Nature Portfolio wishes to improve the reproducibility of the work that we publish. This form provides structure for consistency and transparency in reporting. For further information on Nature Portfolio policies, see our Editorial Policies and the Editorial Policy Checklist.

## Statistics

For all statistical analyses, confirm that the following items are present in the figure legend, table legend, main text, or Methods section.

| n/a | Confirmed | |
|---|---|---|
| ☐ | ☒ | The exact sample size (*n*) for each experimental group/condition, given as a discrete number and unit of measurement |
| ☐ | ☒ | A statement on whether measurements were taken from distinct samples or whether the same sample was measured repeatedly |
| ☒ | ☐ | The statistical test(s) used AND whether they are one- or two-sided<br>*Only common tests should be described solely by name; describe more complex techniques in the Methods section.* |
| ☒ | ☐ | A description of all covariates tested |
| ☒ | ☐ | A description of any assumptions or corrections, such as tests of normality and adjustment for multiple comparisons |
| ☐ | ☒ | A full description of the statistical parameters including central tendency (e.g. means) or other basic estimates (e.g. regression coefficient) AND variation (e.g. standard deviation) or associated estimates of uncertainty (e.g. confidence intervals) |
| ☒ | ☐ | For null hypothesis testing, the test statistic (e.g. *F*, *t*, *r*) with confidence intervals, effect sizes, degrees of freedom and *P* value noted<br>*Give P values as exact values whenever suitable.* |
| ☒ | ☐ | For Bayesian analysis, information on the choice of priors and Markov chain Monte Carlo settings |
| ☒ | ☐ | For hierarchical and complex designs, identification of the appropriate level for tests and full reporting of outcomes |
| ☒ | ☐ | Estimates of effect sizes (e.g. Cohen's *d*, Pearson's *r*), indicating how they were calculated |

*Our web collection on statistics for biologists contains articles on many of the points above.*

## Software and code

Policy information about availability of computer code

| | |
|---|---|
| Data collection | Gel imaging: Amersham Imager 600, Amersham Typhoon; Isothermal titration calorimetry: MicroCal AutoITC200; SPR: Cytiva Biacore T200; CryoEM: 300 kV Titan Krios G3i (TFS) transmission electron microscope equipped with K3 detector (Gatan) and a post-column BioQuantum Imaging filter (Gatan) with a slit width set to 20 eV |
| Data analysis | Assay Analysis: GraphPad Prism v9.2.0; Model Building: COOT v0.8.9.1, Phenix.refine v1.17.1, cryoSPARC v4.2.1, MicroCal PEAQ ITC v.141 |

For manuscripts utilizing custom algorithms or software that are central to the research but not yet described in published literature, software must be made available to editors and reviewers. We strongly encourage code deposition in a community repository (e.g. GitHub). See the Nature Portfolio guidelines for submitting code & software for further information.

## Data

Policy information about availability of data

All manuscripts must include a data availability statement. This statement should provide the following information, where applicable:

- Accession codes, unique identifiers, or web links for publicly available datasets
- A description of any restrictions on data availability
- For clinical datasets or third party data, please ensure that the statement adheres to our policy

The X-ray crystallography data have been deposited in the RCSB with accession codes 9D1I (KLHDC3-EloB/C bound to ubiquitin), 9D1Y (KLHDC3-EloB/C bound to G75R ubiquitin), 9D1Z (KLHDC3-EloB/C bound to G75Q ubiquitin), 9D8P (focused map: UB-KLHDC10-EloB/C) and in the Electron Microscopy Data Bank with codes EMDB: EMD-46644 (consensus map:ARIH1-diUB-neddylated-CRL2KLHDC10) and EMDB: EMD-46645 (focused map:UB-KLHDC10-EloB/C). All other data generated

for Tables, Figures, and Supplementary Figures are available in the Source data file.

## Research involving human participants, their data, or biological material

Policy information about studies with human participants or human data. See also policy information about sex, gender (identity/presentation), and sexual orientation and race, ethnicity and racism.

| | |
|---|---|
| Reporting on sex and gender | N/A |
| Reporting on race, ethnicity, or other socially relevant groupings | N/A |
| Population characteristics | N/A |
| Recruitment | N/A |
| Ethics oversight | N/A |

Note that full information on the approval of the study protocol must also be provided in the manuscript.

# Field-specific reporting

Please select the one below that is the best fit for your research. If you are not sure, read the appropriate sections before making your selection.

[x] Life sciences   [ ] Behavioural & social sciences   [ ] Ecological, evolutionary & environmental sciences

For a reference copy of the document with all sections, see nature.com/documents/nr-reporting-summary-flat.pdf

# Life sciences study design

All studies must disclose on these points even when the disclosure is negative.

| | |
|---|---|
| Sample size | Sample size calculations were not performed. Selected sample sizes were designed to ensure clear and reliable interpretation of the results. Based on previous experience in terms of variability, at least two independent replicates were carried out for all functional assays. |
| Data exclusions | No data were excluded. |
| Replication | All experiments were performed at least twice, with numerous controls. All attempts at replication were successful. |
| Randomization | No grouped samples. |
| Blinding | No grouped samples. |

# Reporting for specific materials, systems and methods

We require information from authors about some types of materials, experimental systems and methods used in many studies. Here, indicate whether each material, system or method listed is relevant to your study. If you are not sure if a list item applies to your research, read the appropriate section before selecting a response.

## Materials & experimental systems

| n/a | Involved in the study | |
|---|---|---|
| x | | Antibodies |
| | x | Eukaryotic cell lines |
| x | | Palaeontology and archaeology |
| x | | Animals and other organisms |
| x | | Clinical data |
| x | | Dual use research of concern |
| x | | Plants |

## Methods

| n/a | Involved in the study | |
|---|---|---|
| x | | ChIP-seq |
| x | | Flow cytometry |
| x | | MRI-based neuroimaging |

# Eukaryotic cell lines

Policy information about cell lines and Sex and Gender in Research

| | |
|---|---|
| Cell line source(s) | Tni (Trichoplusia ni) Insect cells: Expression Systems (94-002S) |
| Authentication | Cell lines were not authenticated. |
| Mycoplasma contamination | Cells were periodically tested for Mycoplasma contamination and were always negative. |
| Commonly misidentified lines (See ICLAC register) | No commonly misidentified cell lines were used in this study. |
